# Structured medication reviews for patients with polypharmacy in primary care: a cross-sectional study in North West London, UK

**DOI:** 10.1177/20542704251325056

**Published:** 2025-04-01

**Authors:** Linwei Li, Geva Greenfield, Benedict W J Hayhoe, Derryn Lovett, Vesselin Novov, Azeem Majeed, Paul Aylin, Hadar Zaman, Thomas Woodcock

**Affiliations:** 1 Department of Primary Care and Public Health, School of Public Health, 4615 Imperial College London, London W12 0BZ, UK; 2 School of Pharmacy and Medical Sciences, Faculty of Life Sciences, 1905 University of Bradford, Bradford, UK; 3 North Cumbria Integrated Care NHS Foundation Trust, 156645 Cumberland Infirmary, Carlisle, UK

**Keywords:** patients, quantitative research, medical error/patient safety, medical management

## Abstract

**Objectives:**

To identify the number and characteristics of patients with polypharmacy receiving structured medication reviews (SMRs) and medication reviews in primary care in 2022, and to evaluate whether the provision of these services is equitable across different demographic and socio-economic groups.

**Design:**

Cross-sectional study.

**Setting:**

Primary care networks in North West London, UK.

**Participants:**

Adults registered with a general practitioner (GP) and regularly prescribed at least five medicines or more.

**Main outcome measures:**

Receipt of at least one SMR and any kind of medication review during the study period (2022).

**Results:**

Among 515,042 adults regularly prescribed with medication, 167,482 were regularly prescribed at least five medicines, defined as polypharmacy. 53.3% (89,220) of these patients received at least one kind of medication review and 17.2% (11,954) of them received SMRs. Patients who were males, black, more affluent, and frailer, were more likely to receive medication reviews, while those who were males, less affluent, and frailer, were more likely to receive SMRs.

**Conclusions:**

Although polypharmacy was common in North West London, only about half of eligible patients received medication reviews, and only 17.2% received SMRs. Different distributions of medication reviews and SMRs by demographic and socio-economic characteristics may indicate inequities in the provision of these services. Policy makers should consider effective ways to incentivise the equitable provision of SMRs.

With the increasing prevalence of multi-morbidity, polypharmacy, defined as routinely using at least five medications,^1^ has become more common. The safe and appropriate management of polypharmacy is a primary obstacle to enhancing medication safety globally. In a UK study, patients who were prescribed five or more medications had an annual medication error rate of 30.1%,^2^ which can result in patient harms such as hospitalisation, disability or even death.^3^ Additionally, non-adherence to medications is relatively common among patients receiving polypharmacy.^4^ Beyond the possible patient risks, polypharmacy might also increase healthcare system costs. An economic analysis in England estimated that annual costs of avoidable adverse drug events was more than £98 million, consuming 181,626 hospital bed-days, and causing 1708 deaths annually.^5^ The World Health Organization (WHO) identified polypharmacy as one of the key action areas in the Strategic Framework of the Global Patient Safety Challenge.^3^

 Medication review, defined as ‘a structured evaluation of a patient's medicines with the aim of optimising medicines use and improving health outcomes’, is widely applied to address polypharmacy.^6^ Some studies indicate that medication reviews can help pharmacists better identify and decrease medication-related problems.^7,8^ In October 2020, NHS England recommended primary care networks (PCNs) offer structured medication reviews (SMRs) to as many potential beneficiary patients as possible given the available clinical capacity.^9^ As a specific type of medication review, SMRs are defined as ‘an evidence-based and comprehensive review of a patient's medication, taking into consideration all aspects of their health’.^9^ SMR is expected to be led by clinical pharmacists, while for some complex cases, it requires multidisciplinary collaboration across the PCN team.^9^ Unlike other medication reviews, which may only focus on certain specific medications or conditions, SMR must consider all the medicines a patient is taking or using.^10^ Additionally, SMR emphasises patient's participation, aiming to provide a personalised approach through a shared decision-making conversation.^11^ SMR is considered an ideal tool to address overprescribing and polypharmacy,^12^ with patients experiencing complex and problematic polypharmacy prioritised for this service.^9^

Several studies have been conducted on medication reviews in the UK.^13–15^ However, there is limited research available regarding the implementation of SMRs.^16,17^ Existing research has high risks of bias due to the selection of study population and the small sample size.^17^ In addition, the equity of providing medication reviews has been neglected in research. Only one qualitative study in England explored the accessibility of medication reviews from the perspective of marginalised patient groups.^18^ Even among the population prioritised for SMRs, there might be disadvantaged groups facing barriers in accessing this service due to socio-economic and demographic factors.

To fill the gap in existing quantitative research, our study focuses on identifying the proportion and characteristics of patients with polypharmacy who receive SMRs and medication reviews in primary care in England. Additionally, we aim to evaluate whether the provision of these services is equitable across different demographic and socio-economic groups.

## Methods

### Data source

This cross-sectional study used data from the Whole Systems Integrated Care (WSIC) database in 2022 to examine the implementation of SMRs and medication reviews, as well as the characteristics of patients receiving these services. The WSIC database records healthcare activities of 2.4 million residents, covering over 95% of the population in North West London (NWL).^19^ As most prescribing activity takes place in general practice in the UK, we decided to focus on prescribing and medication review activity in primary care.

### Study population

The study population were patients regularly prescribed at least five or more medications^1^ during 2022. We included all adults registered with a GP in NWL during the study period. The SNOMED notions of a drug ‘disposition’, which refers to the function of the drug in the body, and of ‘dose form’, were applied to count drug products, and to determine which products to choose. For each patient, we counted the number of distinct, regularly prescribed, dispositions. There were four exclusion criteria: (i) certain non-oral medications, such as powder and suspension for injections, gels, and ointments; (ii) multivitamins; (iii) certain products with no disposition, such as ingredients classified as thickeners, vaccines, or topical medications; (iv) non-regular dispositions that were prescribed on fewer than three occasions within the study period. Prescriptions of the same disposition occurring within 10 days were counted as a single occasion for this purpose. The full list of excluded dose forms can be found in Supplementary Tables 6 and 7 of Woodcock *et al.* (2024).^19^ Individual patient consent was not required as this study used routinely collected de-identified healthcare data.

### Variables

This study set two binary variables as outcomes: whether a patient specifically received at least one SMR, and whether they received any kind of medication review during the study period (2022). We used SNOMED codes to extract information on the provision of medication reviews and SMRs and identified 58 SNOMED concepts describing medication review (Appendix 2). We excluded codes associated with extremely rare types of medication review, retaining the 15 most frequently occurring codes, accounting for 99.42% of all medication reviews conducted in 2022. We further excluded ‘synchronisation of repeat medication’ as it did not fulfil the definition of a medication review. The remaining 14 codes were used to define medication review (Appendix 3).

To examine the characteristics and factors of patients receiving medication reviews and SMRs, we included five covariates, which are classified as demographic characteristics, socio-economic characteristics, and clinical factors (Table 1). Polypharmacy is also a component of the electronic frailty index (eFI), defined as prescription of ≥5 prescribed medications; we excluded this from our analysis in favour of the definition of polypharmacy we used in this research.^19^ In the descriptive analysis, age and eFI scores were converted to categorical variables based on quartiles and literature,^20^ respectively.

**Table 1.** Predictors in the regression model.

Category	Predictor	Description
Demographic characteristics	Age	Continuous variable (years)
	Gender	Female, Male
	Ethnicity	White, Asian or Asian British, Black or Black British, Mixed, Other
Socio-economic characteristics	Index of multiple deprivation (IMD)	Quintiles of IMD where ‘1’ represents the most deprived and ‘5’ represents the least deprived^21^
Clinical factors	Electronic frailty index (eFI)	Continuous variable A higher score indicates greater frailty^20^

### Statistical analysis

First, we described the patients’ characteristics overall in addition to whether they received medication reviews and SMRs. We performed Chi-squared tests to examine the differences in the proportion of all categorical variables between different patient groups. We fitted logistic regression models to analyse the characteristics of patients who underwent medication reviews and SMRs, as well as assess the equity of the delivery of medication reviews and SMRs. Two regression models (Model 1 and Model 2) were fitted, for different outcomes. Model 1 examined the characteristics of patients with polypharmacy related to receiving SMRs, and Model 2 examined the relationships of these patients’ characteristics to the broader outcome of receiving any form of medication review (MR). Variance inflation factors (VIFs) were used to assess multicollinearity after building each model.

Data extraction was conducted via SQL. All statistical analyses were performed in R version 4.2.1, using the readr, gtsummary, tidyverse and car packages.^22^

## Results

### Characteristics of the study population

**Figure 1.**
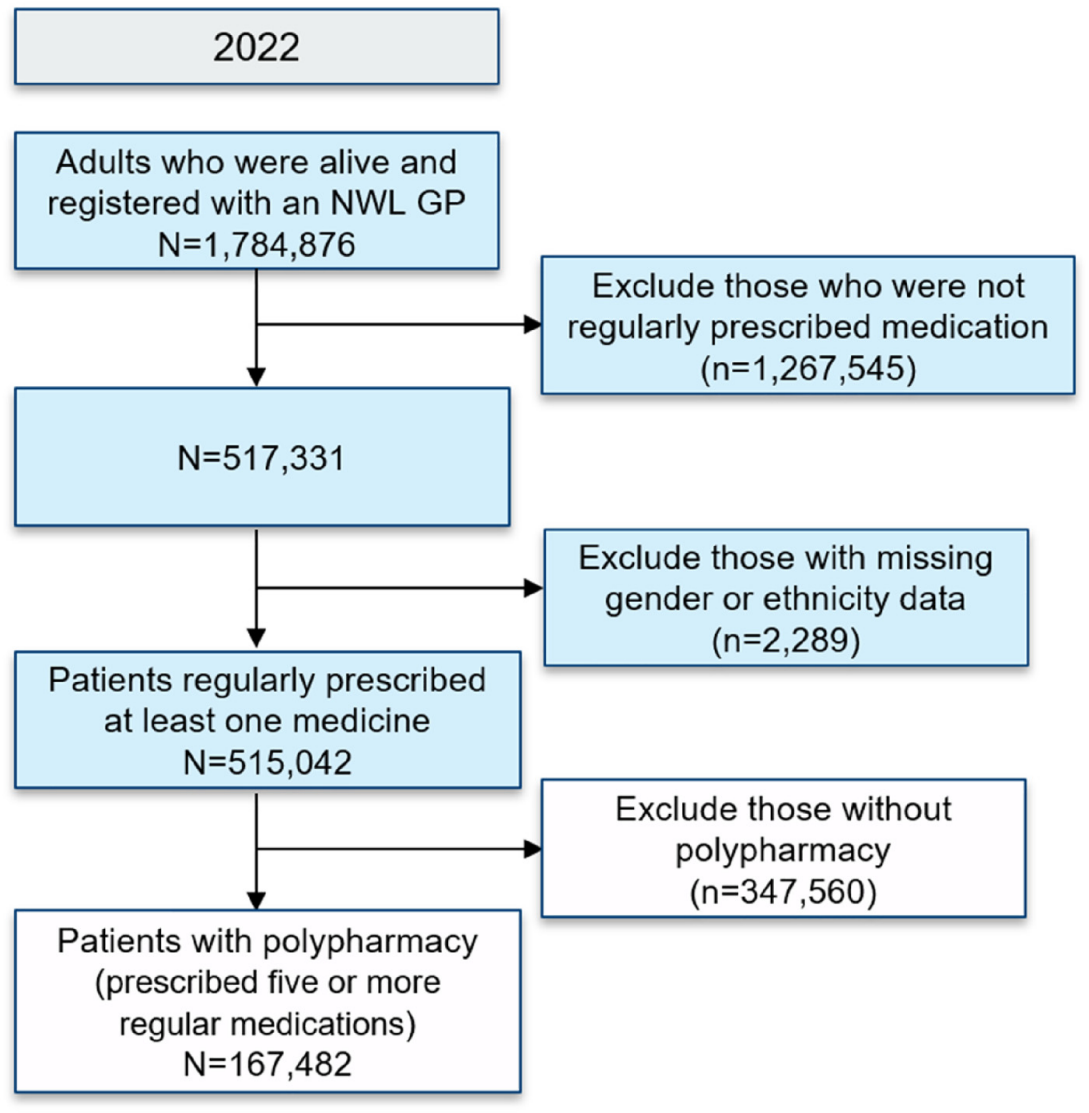
Flow chart describing patient inclusion and total sample size.

There were 1,784,876 adults who met the inclusion criteria, of whom 167,482 (9.4%) had polypharmacy (Figure 1).

Among the 167,482 adults with polypharmacy, 52.4% were females, most patients were over 60 years old (73.3%), and 45.3% were of white ethnicity (Table 2). Nearly half were in the two most deprived quintiles (46.6%), and 39.9% were at least moderately frail.

**Table 2.** Characteristics of the patients with polypharmacy by whether receiving SMRs and medication review of any kind in 2022 (*n* = 167,482).

Variables	Overall *n* = (%)	Receiving structured medication review (SMR)			Receiving medication review of any kind (MR)		
		Yes *n* = (%)	No *n* = (%)	P-value	Yes *n* = (%)	No *n* = (%)	P-value
**Gender**				0.098			<0.001
Female	87,771 (52.4)	15,190 (17.3)	72,581 (82.7)		47,355 (54.0)	40,416 (46.0)	
Male	79,711 (47.6)	13,551 (17.0)	66,160 (83.0)		41,865 (52.5)	37,846 (47.5)	
**Age**				<0.001			<0.001
18–46	11,577 (6.9)	1641 (14.2)	9936 (85.8)		6227 (53.8)	5350 (46.2)	
47–59	33,108 (19.8)	4894 (14.8)	28,214 (85.2)		17,300 (52.3)	15,808 (47.7)	
60–71	51,422 (30.7)	8068 (15.7)	43,354 (84.3)		26,710 (51.9)	24,712 (48.1)	
72+	71,375 (42.6)	14,138 (19.8)	57,237 (80.2)		38,983 (54.6)	32,392 (45.4)	
**Ethnicity**				<0.001			<0.001
White	75,895 (45.3)	13,480 (18.2)	62,055 (81.9)		40,694 (53.6)	35,201 (46.4)	
Asian or Asian British	58,404 (34.9)	8805 (15.1)	49,599 (84.9)		31,565 (54.0)	26,839 (46.0)	
Black or Black British	16,038 (9.6)	3103 (19.3)	12,935 (80.7)		8842 (55.1)	7196 (44.9)	
Mixed	4215 (2.5)	764 (18.1)	3451 (81.9)		2237 (53.1)	1978 (46.9)	
Other	12,930. (7.7)	2229 (17.2)	10,701 (82.8)		5882 (45.5)	7048 (54.5)	
**IMD**				<0.001			<0.001
1 (Most deprived)	23,775 (14.2)	4795 (20.2)	18,980 (79.8)		12,041 (50.6)	11,734 (49.9)	
2	54,280 (32.4)	9325 (17.2)	44,955 (82.8)		27,635 (50.9)	26,645 (49.1)	
3	48,366 (28.9)	8474 (17.5)	39,892 (82.5)		25,769 (53.3)	22,597 (46.7)	
4	28,121 (16.8)	4516 (16.1)	23,605 (83.9)		15,816 (56.2)	12,305 (43.8)	
5 (Least deprived)	12,940 (7.7)	1631 (12.6)	11,309 (87.4)		7959 (61.5)	4981 (38.5)	
**eFI score**				<0.001			<0.001
Fit	36,349 (21.7)	3736 (10.3)	32,613 (89.7)		16,578 (45.6)	19,771 (54.4)	
Mild frailty	64,372 (38.4)	8663 (13.5)	55,709 (86.5)		32,924 (51.1)	31,448 (48.9)	
Moderate frailty	45,029 (26.9)	9356 (20.8)	3,5673 (79.2)		25,890 (57.5)	19,139 (42.5)	
Severe frailty	21,732 (13.0)	6986 (32.1)	14,746 (67.9)		13,828 (63.6)	7904 (36.4)	

*Note*: *p*-Value derived from Chi-squared statistic testing for differences in the proportion across whether receiving SMRs and medication review of any kind.

### Patients receiving SMRs or medication review of any kind

Of 167,482 patients with polypharmacy, 89,220 (53.3%) received a medication review of any kind (MR, including those who received an SMR) during the study period (2022). 28,741 received at least one SMR, only accounting for 17.2% of the study population. There was a statistically significant association between each predictor and outcome (*p* < 0.001), except between gender and receiving SMRs (*p* = 0.098). The very slight difference in proportion of females (17.3%) and males (17.0%) receiving SMRs was not significant, while there was a small but significant difference in proportion of females receiving MR (54.0%) and that of males (52.5%). There was no directional pattern in the small differences in proportion receiving MRs by age band. There was, however, a clear increasing trend in proportion receiving SMRs with increasing age (from 14.2% of 18–46-year-olds to 19.8% of those aged 72 or older). A higher proportion of Black patients received both SMRs (19.3%) and MRs (55.1%) compared with other ethnicities. A lower proportion of Asian patients received SMRs (15.1%), and a lower proportion of patients of other ethnicities received MRs (45.5%), compared with other ethnicities. Whilst the proportion receiving SMRs increased with socio-economic deprivation (12.6% among those living in the least deprived quintile areas, to 20.2% in the most deprived quintile), this pattern was reversed for MRs (61.5% in the least deprived quintile to 50.6% in the most deprived quintile). For both outcomes, reviews were more frequent as frailty increased (10.3% of ‘fit’ individuals to 32.1% of those with ‘severe frailty’ for SMRs, and 45.6%–63.6% for MRs).

### Factors associated with receiving medication reviews and SMRs

There were significant associations between each included variable and the outcome of receiving SMRs (Table 3, Model 1). Independently of other variables, each additional year of age was associated with a reduction of 1% in odds of SMR (OR 0.99, 95% CI: 0.99–0.99). Male gender (OR 1.15, 95% CI: 1.12–1.18) and living in a more deprived area (OR 1.63, 95% CI: 1.53–1.73) were associated with higher odds of receiving SMRs. For every 1% increase in eFI, odds of SMR increased by 4% (OR 1.04, 95% CI: 1.04–1.04). Compared with White patients, Asian patients (OR 0.78, 95% CI: 0.77 to 0.80) were less likely to undergo SMRs. No significant association was observed for black, mixed ethnicity or other ethnicities.

**Table 3.** Logistic regression results of two outcomes for patients with polypharmacy based on the WSIC data of NWL in 2022 (*n* = 167,482).

Variables	Receiving SMRs (Model 1)			Receiving medication review of any kind (Model 2)		
	AOR	95% CI	P-value	AOR	95% CI	P-value
**Age (years)**	0.99	0,99, 0.99	< 0.001	0.99	0.99, 0.99	<0.001
**Gender**						
Female(reference)	–	–		–	–	
Male	1.15	1.12, 1.18	<0.001	1.02	1.00, 1.04	0.040
**Ethnicity**						
White(reference)	–	–		–	–	
Asian or Asian British	0.78	0.77, 0.80	<0.001	1.02	0.99, 1.04	0.2
Black or Black British	1.02	0.98, 1.07	0.292	1.12	1.08, 1.16	<0.001
Mixed	0.99	0.92, 1.08	0.901	1.01	0.95, 1.08	0.8
Other	0.97	0.92, 1.02	0.242	0.75	0.72, 0.77	<0.001
**IMD**						
5 (Least deprived) (reference)	–	–		–	–	
4	1.33	1.25, 1.42	<0.001	0.78	0.75, 0.82	<0.001
3	1.48	1.39, 1.57	<0.001	0.67	0.64, 0.70	<0.001
2	1.42	1.34, 1.50	<0.001	0.60	0.57, 0.62	<0.001
1 (Most deprived)	1.63	1.53, 1.73	<0.001	0.57	0.55, 0.60	<0.001
**EFi_score**	1.04	1.04, 1.04	<0.001	1.03	1.03, 1.03	<0.001

In relation to the broader outcome of receiving MRs (Table 3, Model 2), the same pattern was seen in relation to age and eFI. There were some differences in the associations with other variables, compared with SMR. Males (OR 1.02, 95% CI: 1.00–1.04) were only slightly more likely to receive MRs than females. Regarding ethnicity, black patients (OR 1.12, 95% CI: 1.08–1.16) were associated with higher odds of MRs, while other ethnicities (OR 0.75, 95% CI: 0.72–0.77) were less likely to receive MRs. Higher levels of socio-economic deprivation were associated with lower odds of MRs, and participants in the most deprived quintile (OR 0.57, 95% CI: 0.55–0.60) had 43% lower odds of MRs versus those in the least deprived quintile. The difference between the multivariable analysis and univariate results on association with gender can be explained by the different distribution of frailty among males and females, with the mean number of eFI components being 7.48 for males and 8.75 for females. As SMRs are more likely to be provided to frailer patients, the rate of females receiving SMRs is higher than that of males in the univariate analysis. After adjusting for other variables, including frailty, males have slightly higher odds of receiving SMRs.

After building the regression models, VIF was used to examine multicollinearity of the predictors. The VIF of each predictor in all these models was less than two, indicating no significant collinearity.

## Discussion

We found a lower than anticipated number of SMRs being undertaken in primary care. 167,482 adults were regularly prescribed at least five medicines (defined in this study as polypharmacy) in NWL in 2022. More than half of adults with polypharmacy received some form of medication review; however, only 17.2% of patients with polypharmacy received a SMR during 2022. Patients with polypharmacy were more likely to receive medication reviews if they were males, black, more affluent, and frailer; and more likely to receive a SMR if they were males, white, less affluent, and frailer.

In 2022, 167,482 adults (9.4%) were identified as having polypharmacy in NWL, nearly one-third of those taking regular medications. National research in England similarly illustrated the severity of this issue, finding over 876,000 people (1.5%) took at least ten medicines in England in 2022.^23^ The proportion of patients with polypharmacy receiving SMRs is low, at only 17.2% in our study. Limited clinical capacity might restrict implementation of medication reviews and SMRs, especially if this is not seen as a PCN priority. Another barrier to implementation might be clinicians lack of awareness of recommendations on use of SMRs.^17^ A qualitative study found that some pharmacists might not fully comprehend the differences between SMRs and other medication reviews and could not get enough support from GP colleagues.^17^ New clinical coding requirements for SMRs were released in 2020, but many factors affect clinical coding quality,^24^ potentially weakening effectiveness of electronic health records in assessing implementation of SMRs.

Significant differences were found in the likelihood of receiving medication reviews and SMRs by gender, ethnicity, and IMD, revealing the provision of medication reviews and SMRs was not equitable. Disparities in disease distribution and healthcare engagement^21,25,26^ between different demographic and socio-economic groups might explain this unequal distribution. For example, males have higher risk of cardiovascular diseases and take more cardiovascular drugs, which are prone to causing harmful drug interactions,^26^ this could explain why males were more likely to undergo medication reviews compared with females. Since accessibility to services is one component of IMD,^21^ people living in the most deprived areas have more barriers to accessing services including healthcare, hindering their ability to receive medication reviews. Whilst those living in an area of high deprivation were more likely to receive an SMR, since the majority of reviews conducted were not SMRs, overall the effect is still to render this group less likely to have their medications reviewed than those living in more affluent areas. The underlying reason for this difference in trends for the two outcomes is not clear from the results of this study, and further research is needed to understand the reasons behind these differences.

### Implications for practice and policy/future research

Based on our findings, we suggest two key policy action areas: increasing coverage of SMRs for prioritised groups and improving equity in the provision of SMRs among these groups. To achieve these goals, potential solutions include encouraging GPs and pharmacists to provide SMR services and employing innovative approaches, such as remote consultations, to enhance accessibility of SMRs.

Acknowledging the limited capacity of primary care clinicians, a potential solution might be motivating GPs and pharmacists to proactively offer SMRs to patients who may benefit through incentive measures. Previous evidence indicated the effectiveness of paying healthcare providers for performance. For example, the Quality and Outcomes Framework (QOF) incentivises GP practices through financial reward for achievement of clinical quality in a range of areas; medication reviews were included in QOF in 2004 but were removed in 2013.^27^

Since accessibility is one of the primary barriers leading to inequities in the utilisation of healthcare services,^21,25^ the NHS England guidance for SMRs explored innovative strategies to address this issue. For example, clinicians are suggested to participate in appropriate training programmes to ensure they can conduct remote SMRs.^10^

Finally, it is essential to accurately record medication reviews and SMRs in electronic medical records using appropriate clinical codes. NHS England guidance for SMRs was published in September 2020 as a reference for PCNs.^10^ According to the guidance, it is necessary to set qualifications and assessments for clinicians and ask them to enrol in training programmes.^10^

### Strengths and limitations

To our knowledge this is the first large quantitative study investigating implementation of SMRs after their recommendation by NHS England. The large sample size and representativeness of the WSIC dataset supports strong external validity of the results. In contrast to previous studies collecting data from several GPs in a certain area,^13,17^ the sample of this study was inclusive and generalisable both on the regional and national levels, reducing potential selection bias. Additionally, this study used a robust method to identify patients with polypharmacy. Different from previous studies that only focused on the number of medicines, our approach used the concepts of ‘drug disposition’ and ‘regular medications’ to define polypharmacy.^19^ This method is deemed more reflective of the patient's prescribed conditions and medicine functions within the body.

This study has some limitations. Although NHS England set prioritised groups for SMRs, the actual number of SMRs provided is limited by the clinical pharmacist capacity of PCNs.^11^ However, the WSIC dataset does not include information on PCN characteristics such as staffing levels, and we were therefore unable to account for this in our analysis. This does not, however, detract from the facts of the variation in delivery of SMRs identified in this study. In addition, data were routinely collected through delivery of primary care, thereby depending on the quality of primary healthcare staff's coding practices. In 2020, NHS England required that PCNs should clearly record all SMRs within GP IT systems.^28^ Besides, practices are advised to use designated clinical codes to ensure payment,^28^ which could motivate them to code SMR and medication reviews correctly. Therefore, this issue is unlikely to introduce significant bias into our results. Finally, we used an established definition of polypharmacy which did not account for further complexity of polypharmacy, or for problematic prescribing. It is therefore possible that some of the variation found is associated with details of the specific drugs prescribed to patients, and this should be explored in future studies.

## Conclusion

This study provides insights into the implementation of SMRs in NWL, indicating that significant improvements are needed to realise the vision set out by NHS England. Policy makers should consider ways in which SMRs can be incentivised to improve their use, particularly with priority groups such as those with polypharmacy. Additionally, even among patients with polypharmacy, we found potentially inequitable provision of SMRs across different demographic and socio-economic groups, highlighting the need for equity-focused interventions. Future research should carry out a longitudinal evaluation of SMRs to explore implementation and effectiveness over time. Meanwhile, similar studies should be conducted to understand variation in service delivery between GP practices, and among other priority groups for SMRs, such as patients with severe frailty.

## Supplemental Material

sj-docx-1-shr-10.1177_20542704251325056 - Supplemental material for Structured medication reviews for patients with polypharmacy in primary care: a cross-sectional study in North West London, UK Supplemental material, sj-docx-1-shr-10.1177_20542704251325056 for Structured medication reviews for patients with polypharmacy in primary care: a cross-sectional study in North West London, UK by Linwei Li, Geva Greenfield, Benedict W J Hayhoe, Derryn Lovett, Vesselin Novov, Azeem Majeed, Paul Aylin, Hadar Zaman and Thomas Woodcock in JRSM Open

sj-docx-2-shr-10.1177_20542704251325056 - Supplemental material for Structured medication reviews for patients with polypharmacy in primary care: a cross-sectional study in North West London, UK Supplemental material, sj-docx-2-shr-10.1177_20542704251325056 for Structured medication reviews for patients with polypharmacy in primary care: a cross-sectional study in North West London, UK by Linwei Li, Geva Greenfield, Benedict W J Hayhoe, Derryn Lovett, Vesselin Novov, Azeem Majeed, Paul Aylin, Hadar Zaman and Thomas Woodcock in JRSM Open
