# Supplementary material for: Structured medication reviews for patients with polypharmacy in primary care: a cross-sectional study in North West London, UK
Source: JRSM Open. 2025 Apr 1;16(4):20542704251325056. doi: 10.1177/20542704251325056 (PMC11963712; doi:10.1177/20542704251325056)
Supplement: sj-docx-1-shr-10.1177_20542704251325056 - Supplemental material for Structured medication reviews for patients with polypharmacy in primary care: a cross-sectional study in North West London, UK [file sj-docx-1-shr-10.1177_20542704251325056.docx]

**Appendices**

**Appendix 1**

List of dose forms excluded in defining regularly prescribed medication

| Term name of the dose form | | | |
| --- | --- | --- | --- |
| Conventional release solution for injection (dose form) | Enema (qualifier value) | Conventional release eye gel (dose form) | Irrigation (qualifier value) |
| Conventional release eye drops (dose form) | Conventional release oromucosal solution for mouthwash (dose form) | Paint (qualifier value) | Wash (qualifier value) |
| Cream (basic dose form) | Bath additive (qualifier value) | Conventional release oral gel (dose form) | Modified-release drops (qualifier value) |
| Ointment (basic dose form) | Shampoo (qualifier value) | Conventional release dental gel (dose form) | Powder and suspension for suspension for injection (qualifier value) |
| Prolonged-release transdermal patch (dose form) | Irrigation solution (qualifier value) | Intrauterine device (qualifier value) | Conventional release dental suspension (dose form) |
| Gel (basic dose form) | Conventional release eye ointment (dose form) | Medicated nail lacquer (qualifier value) | Conventional release cutaneous solution (dose form) |
| Conventional release suspension for injection (dose form) | Pessary (basic dose form) | Conventional release intravesical solution (dose form) | Conventional release cutaneous emulsion (dose form) |
| Basic dose form with liquid state of matter (basic dose form) | Foam (basic dose form) | Conventional release vaginal gel (dose form) | Conventional release rectal ointment (dose form) |
| Pressurised inhalation (qualifier value) | Conventional release oromucosal gel (dose form) | Implant (basic dose form) | Prolonged-release suspension for injection (dose form) |
| Dose form not applicable (qualifier value) | Infusion (qualifier value) | Vaginal delivery system (qualifier value) | Conventional release gas for inhalation (dose form) |
| Powder and solvent for solution for injection (qualifier value) | Conventional release nasal drops (dose form) | Ear/eye drops solution (qualifier value) | Conventional release emulsion for injection (dose form) |
| Impregnated dressing (qualifier value) | Stick (basic dose form) | Ear/eye/nose drops solution (qualifier value) | Powder for conventional release suspension for injection (dose form) |
| Powder and solvent for suspension for injection (qualifier value) | Powder for conventional release solution for infusion (dose form) | Conventional release solution for cutaneous spray (dose form) | Pastille (basic dose form) |
| Conventional release ear drops (dose form) | Medicated plaster (qualifier value) | Powder and solvent for prolonged-release suspension for injection (qualifier value) | Powder and solvent for solution for infusion (qualifier value) |
| Powder for conventional release solution for injection (dose form) | Paste (basic dose form) | Conventional release nasal ointment (dose form) |  |

**Appendix 2**

List of medication reviews recorded in SNOMED CT

| Parent | Child | Child-child |
| --- | --- | --- |
| Review of medication | Anticoagulant medication review | Review of international normalised ratio time in therapeutic range |
|  | Asthma medication review |  |
|  | Bisphosphonate medication review |  |
|  | Cardiac medication review |  |
|  | Cardiovascular disorder medication review | Coronary heart disease medication review |
|  |  | Heart failure medication review |
|  |  | Hypertension medication review |
|  | Comprehensive medication therapy review |  |
|  | Concordance and compliance level 2 medication review |  |
|  | Dispensing review of use of medicines | Dispensing review of use of medicines invitation |
|  |  | Dispensing review of use of warfarin |
|  | Endocrine disorder medication review | Diabetes medication review |
|  | Gastrointestinal disorder medication review |  |
|  | Hematologic disorder medication review |  |
|  | High risk drug monitoring review | High risk drug monitoring annual review |
|  |  | High risk drug monitoring monthly review |
|  |  | High risk drug monitoring six monthly review |
|  |  | High risk drug monitoring three monthly review |
|  |  | High risk drug monitoring two monthly review |
|  | Infectious disease medication review | Human immunodeficiency virus medication review |
|  | Medication review by community nurse |  |
|  | Medication review by practice nurse |  |
|  | Medication review of medical notes |  |
|  | Medication review with patient |  |
|  | Medication review without patient |  |
|  | Medicine labeling amended |  |
|  | Mental health medication review | Annual review of lithium therapy |
|  |  | Antipsychotic medication review |
|  |  | Dementia medication review |
|  |  | Depression medication review |
|  | Metabolic disorder medication review | Diabetes medication review |
|  |  | Dyslipidemia medication review |
|  |  | Gout medication review |
|  | Neurological disorder medication review | Epilepsy medication review |
|  | Osteoporosis medication compliance review |  |
|  | Pain medication review |  |
|  | Palliative care medication review |  |
|  | Polypharmacy medication review |  |
|  | Pregnancy and lactation medication review |  |
|  | Pulmonary disorder medication review | Chronic obstructive lung disorder medication review |
|  | Renal disorder medication review |  |
|  | Respiratory disease medication review |  |
|  | Review of current supply of medication |  |
|  | Review of opioid medication |  |
|  | Stopping Over-Medication of People with Learning Disability, Autism or Both medication review |  |
|  | Structured medication review |  |
|  | Synchronization of repeat medication |  |
|  | Targeted medication therapy review |  |

**Appendix 3**

List of medication reviews conducted in 2022

| Name | Occurrence | Name | Occurrence |
| --- | --- | --- | --- |
| **Review of medication** | **175072** | Respiratory disease medication review | 306 |
| **Medication review with patient** | **83216** | High risk drug monitoring review | 183 |
| **Structured medication review** | **55867** | Cardiac medication review | 156 |
| **Asthma medication review** | **49347** | Dementia medication review | 147 |
| **Medication review of medical notes** | **36031** | Medication review by community nurse | 110 |
| **Mental health medication review** | **34573** | STOMP (Stopping Over-Medication of People with Learning Disability, Autism or Both) medication review | 104 |
| Synchronisation of repeat medication | 15364 | Opioid medication review | 70 |
| **Polypharmacy medication review** | **13611** | Coronary heart disease medication review | 69 |
| **Medication review without patient** | **12584** | Chronic obstructive lung disorder medication review | 66 |
| **Diabetes medication review** | **11330** | Medication review by practice nurse | 58 |
| **Depression medication review** | **7934** | Bisphosphonate medication review | 52 |
| **Heart failure medication review** | **5824** | High risk drug monitoring three monthly review | 41 |
| **Anticoagulant medication review** | **5404** | Palliative care medication review | 20 |
| **Concordance and compliance level 2 medication review** | **1074** | High risk drug monitoring annual review | 20 |
| **Dispensing review of use of medicines** | **742** | Osteoporosis medication compliance review | 14 |
| Hypertension medication review | 702 | High risk drug monitoring six monthly review | 7 |
| Epilepsy medication review | 471 | Medicine labelling amended | 1 |
| Antipsychotic medication review | 368 | Lithium annual review | 1 |
